# N7-methylguanosine modification of lncRNAs in a rat model of hypoxic pulmonary hypertension: a comprehensive analysis

**DOI:** 10.1186/s12864-021-08188-8

**Published:** 2022-01-07

**Authors:** Huan Wang, Ren Biao Chen, Si Ni Zhang, Rui Feng Zhang

**Affiliations:** 1 Department of Respiratory Medicine, Zhongda Hospital of Southeast University, 87 Dingjiaqiao Road, Nanjing, 210009 People’s Republic of China; 2 Department of Radiology, Sir Run Run Shaw Hospital, Zhejiang University School of Medicine, Hangzhou, 310000 People’s Republic of China

**Keywords:** Hypoxic pulmonary hypertension, lncRNAs, N7-methylguanosine modification, m7G-sequencing

## Abstract

**Background:**

Long non-coding RNAs (lncRNAs) play a critical role in the pathogenesis of hypoxic pulmonary hypertension (HPH). The role of N7-methylguanosine (m7G) modification in lncRNAs has received increased attentions in recent years. However, the m7G-methylation of lncRNA in HPH has yet to be determined. We have therefore performed a transcriptome-wide analysis of m7G lncRNAs in HPH.

**Results:**

Differentially-expressed m7Gs were detected in HPH, and m7G lncRNAs were significantly upregulated compared with non-m7G lncRNAs in HPH. Importantly, this was the first time that the upregulated m7G lncXR_591973 and m7G lncXR_592398 were identified in HPH.

**Conclusion:**

This study provides the first m7G transcriptome-wide analysis of HPH. Importantly, two HPH-associated m7G lncRNAs were identified, although their clinical significance requires further validation.

**Supplementary Information:**

The online version contains supplementary material available at 10.1186/s12864-021-08188-8.

## Introduction

Pulmonary hypertension (PH) is a lethal disease causing increased vascular resistance and eventually leading to right ventricular failure and death [1]. Hypoxic pulmonary hypertension (HPH) is classified as a group III PH based on pathogenesis. It is a progressive disease induced by chronic hypoxia, which normally results from severe interstitial lung diseases and chronic obstructive pulmonary disease (COPD) [2]. The pathophysiology of HPH is characterized by pulmonary arterial remodelling, mediated via unusual proliferation of pulmonary artery endothelial cells (PAECs) and pulmonary artery smooth muscle cells (PASMCs), but also activation of quiescent fibroblasts [3, 4]. However, the underlying molecular mechanisms remain imperfectly understood due to the complexity and malignancy of HPH.

In recent years, long non-coding RNAs (lncRNAs) have received extensive attention because of their critical role in the regulation of biological phenomena such as cell proliferation, apoptosis, migration and invasion [5–8]. lncRNAs generally refer to a class of transcripts longer than 200 nucleotides (nt) that lack protein-coding ability [9]. They interact with proteins, interfere with microRNA by acting as molecular sponges, modify the epigenome and interfere with gene expression by binding with gene promoters [10–13]. The crucial role of lncRNAs in HPH pathogenesis in idiopathic PH was highlighted by the up-regulation of 2004 lncRNAs and down-regulation of 507 lncRNAs [14]. Subsequently, multiple lncRNAs have been implicated in the pathogenesis of HPH, such as lncRNA MEG3 and lncRNA Tug1. It is noteworthy that their dysregulated expression has been observed in HPH [15–17]. RNA modification plays a critical role in the regulation of gene expression [18]. Nevertheless, post-transcription modification of lncRNAs in HPH remains largely unexplored.

N7-Methylguanosine (m7G) is a ubiquitous post-transcriptional modification of mRNA and lncRNA in eukaryotes, and it is essential for efficient gene expression and cell viability [19, 20]. During transcription, m7G is incorporated at the 5′ end, modulating the various events in the mRNA cycle including RNA splicing, polyadenylation, nuclear export and translation [21]. Although evidence increasingly indicates that m7G modification is closely associated with the initiation and progression of various diseases, the RNA m7G-methylation profile of HPH has yet to be reported. This study provides the first transcriptome-wide analysis of m7G profile in HPH and control group, demonstrating the enormous diversity of m7G modification patterns in these two groups. It is hoped that this study will facilitate further investigation into the potential role of m7G modification in HPH pathogenesis.

## Results

### General features of m7G methylation of lncRNAs in HPH and control samples

**Fig. 1:**
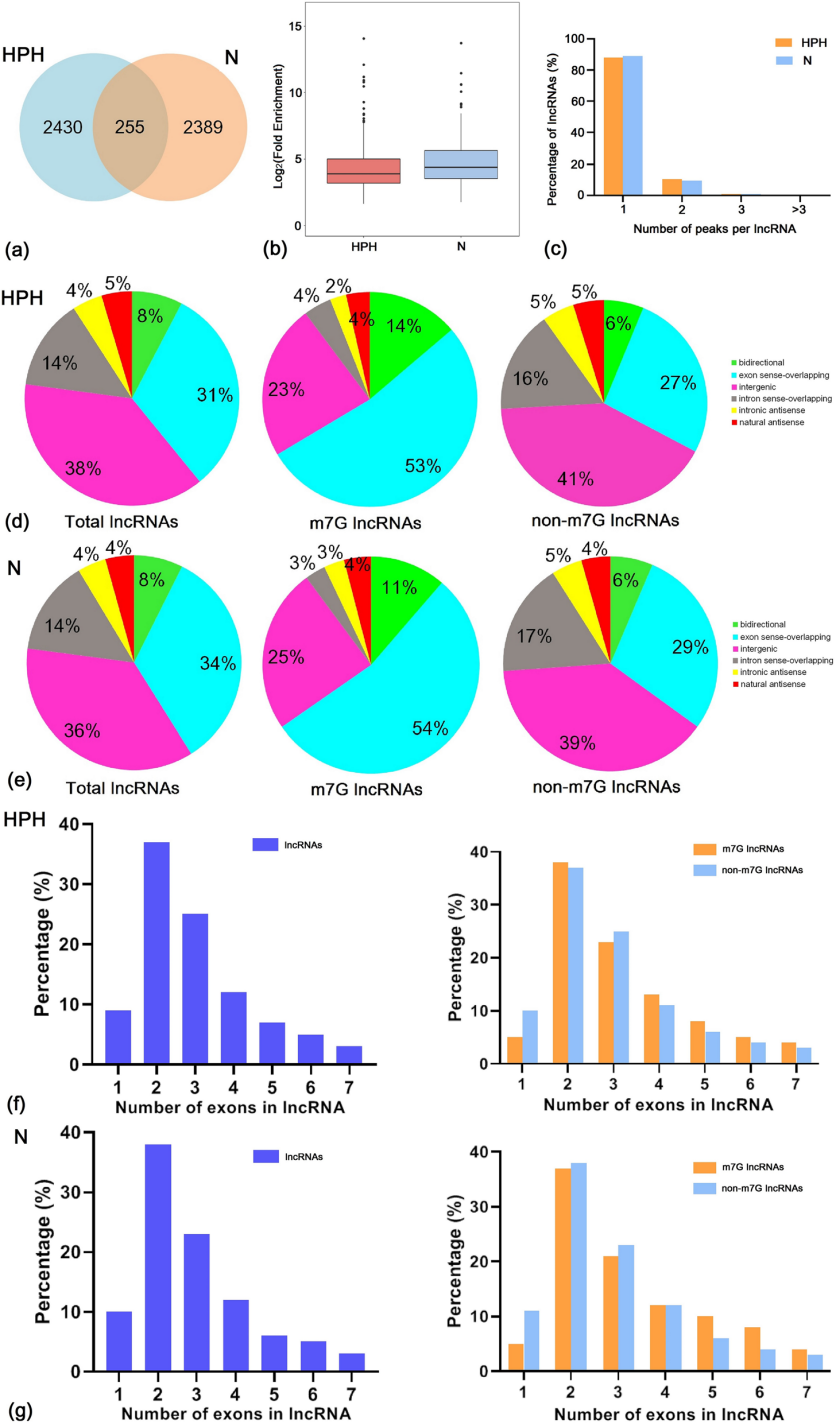
Overview of m7G methylation within lncRNAs in the lungs of HPH and normobaric normoxic rats. **a** Venn diagram showing the number of HPH-unique, normoxic (N)-unique and common m7G peaks of lncRNAs. **b** Boxplot of m7G peaks enrichment in lncRNAs of HPH and N groups. **c** Proportion of lncRNAs with different numbers of m7G peaks in the two groups. **d** and **e** Classification of total lncRNAs, m7G-methylated lncRNAs and non-m7G lncRNAs in the HPH and N groups: proportion of each type is shown in the pipe plot. **f** and **g** Proportion of lncRNAs, m7G-methylated lncRNAs and non-m7G lncRNAs containing different numbers of exons. Up to seven exons are shown. *n* = 3 for each group

A rat model of hypoxia-induced PH was successfully established [22]. Pulmonary tissues from HPH and control (normoxia, N) rats were used for transcriptome-wide m7G-MeRIP-seq and RNA-seq analyses. The HPH tissues revealed 2685 m7G peaks, while the control rat tissues showed 2644 m7G peaks. The HPH and control tissues shared 255 peaks, which accounted for only 5.0% of all peaks in the two groups (Fig. 1a; Additional file 1: Data S1). The low percentage of shared m7G peaks within the lncRNAs indicated differences in the m7G pattern between the two groups. Additionally, the level of m7G in the total lncRNA derived from HPH rats was lower than that in the control group (Fig. 1b).

Analysis of the distribution of m7G peaks in each lncRNA revealed no significant difference between the two groups. Approximately 80% of the modified lncRNAs showed unique m7G peaks, while almost 10% of the modified lncRNAs revealed two m7G peaks (Fig. 1c).

To further elucidate the m7G methylation pattern of lncRNAs in the HPH and control groups, the samples were divided into the following six categories based on the positional relationship between the m7G methylated lncRNAs and mRNAs: bidirectional, exon sense overlap, intron sense overlap, intron antisense overlap, natural antisense overlap, and intergenic overlap. The results revealed that the majority of lncRNAs contained m7G sites with exon sense overlap. In deed, HPH samples accounted for 53%, while control samples constituted 54% of such lncRNAs. Most lncRNAs carried intergenic m7G sites, with HPH samples accounting for 23% and control samples constituting 25% of such lncRNAs. Only approximately 2% of the methylation sites were located in the intronic antisense group (Fig. 1d-e).

Further analysis of the m7G methylated lncRNAs showed that more than 30% of total lncRNA contained two exons in both HPH and control groups (Fig. 1f-g). Similarly, m7G and non-m7G lncRNAs were mainly encoded by two exons, suggesting that the two exons in the HPH and control groups contributed to most of the m7G methylation in lncRNAs.

### Length and distribution of differentially-methylated lncRNAs

**Fig. 2:**
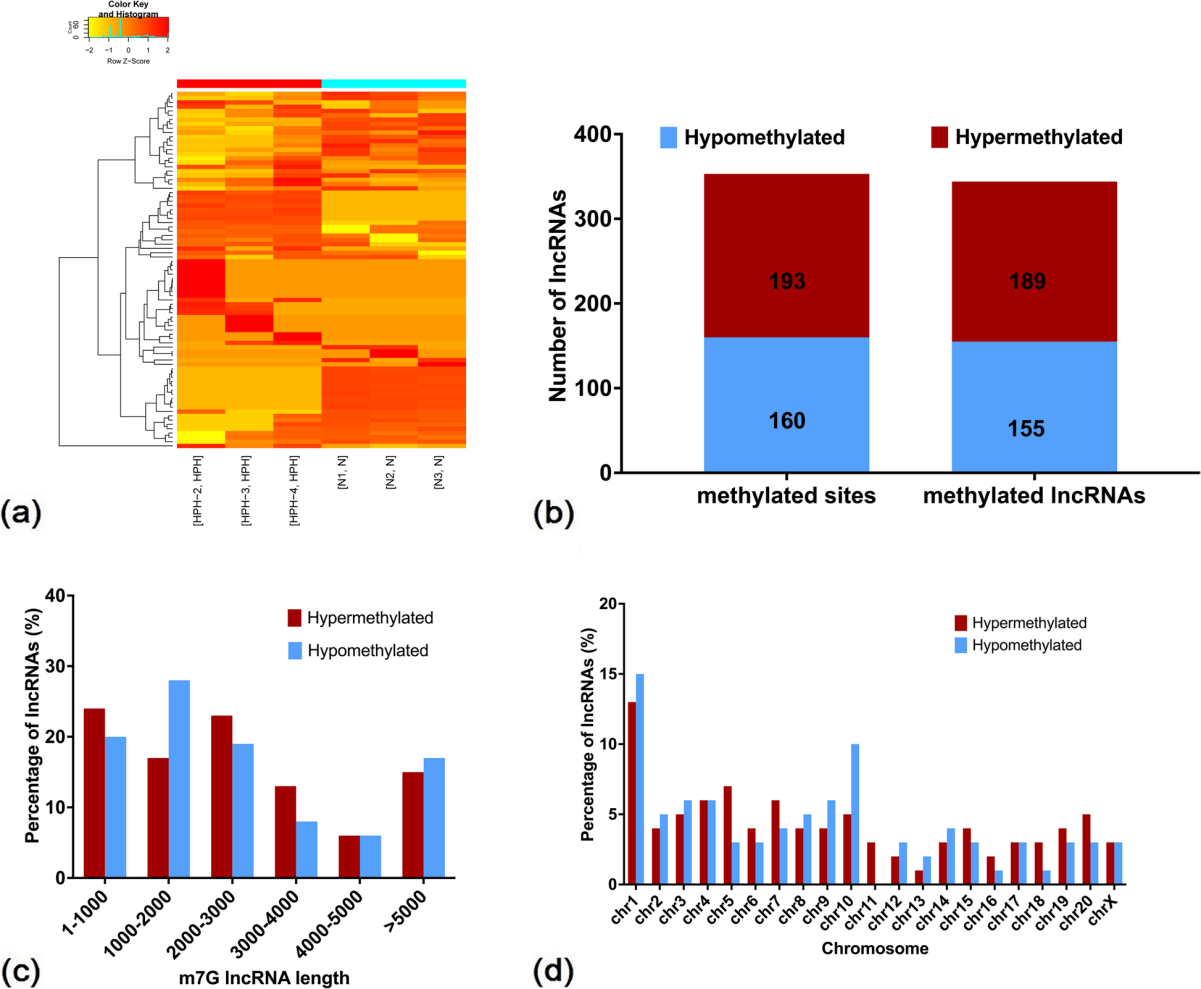
Distribution of lncRNAs with differential m7G modification. **a** Heatmap depicting hierarchical clustering of m7G-altered lncRNAs in the lungs of HPH and normoxic (N) rats. Red represents higher expression and yellow denotes lower expression levels. **b** Numbers of differentially-methylated peaks and associated lncRNAs. **c** Length of differentially m7G-methylated lncRNAs. **d** Distribution of differentially m7G-methylated lncRNAs on chromosomes. *n* = 3 for each group

As displayed in the heatmap of m7G lncRNA expression (Fig. 2a), 353 differentially methylated m7G sites were identified across 344 lncRNAs. Of these, 55% (193/353) were significantly hypermethylated and 45% (160/353) were significantly hypomethylated (fold change ≥2 and *P* ≤ 0.00001; Fig. 2b; Additional file 2: Data S2). The 353 differentially methylated m7G sites were located in 344 lncRNAs, including hypermethylated (189) and hypomethylated (155) types (Fig. 2b).

Analysis of the length of the differentially m7G-methylated lncRNAs revealed differences between hypermethylated and hypomethylated types (Fig. 2c). The length of the hypermethylated m7G lncRNAs was primarily 1–1000 bp (24%) and 2000–3000 bp (23%), while the length of the hypomethylated m7G lncRNAs was mostly in the range of 1000 to 2000 bp (29%).

To analyse the distribution of the differentially m7G-methylated lncRNAs across chromosomes, the enrichment level of m7G methylated lncRNAs on each chromosome was further determined. As shown in Fig. 2d, the hypomethylated m7G lncRNAs were primarily located on chromosomes 1 (13.99%), 5 (7.25%) and 4 (6.74%) and the hypomethylated m7G lncRNAs were primarily located on chromosomes 1 (15.63%), 10 (10.0%) and 3 (6.88%).

### Functional analysis of genes near differentially methylated lncRNAs

To elucidate the role of differentially methylated lncRNAs in the occurrence and development of HPH, GO and KEGG pathway analyses of genes located near differentially methylated lncRNAs were performed.

**Fig. 3:**
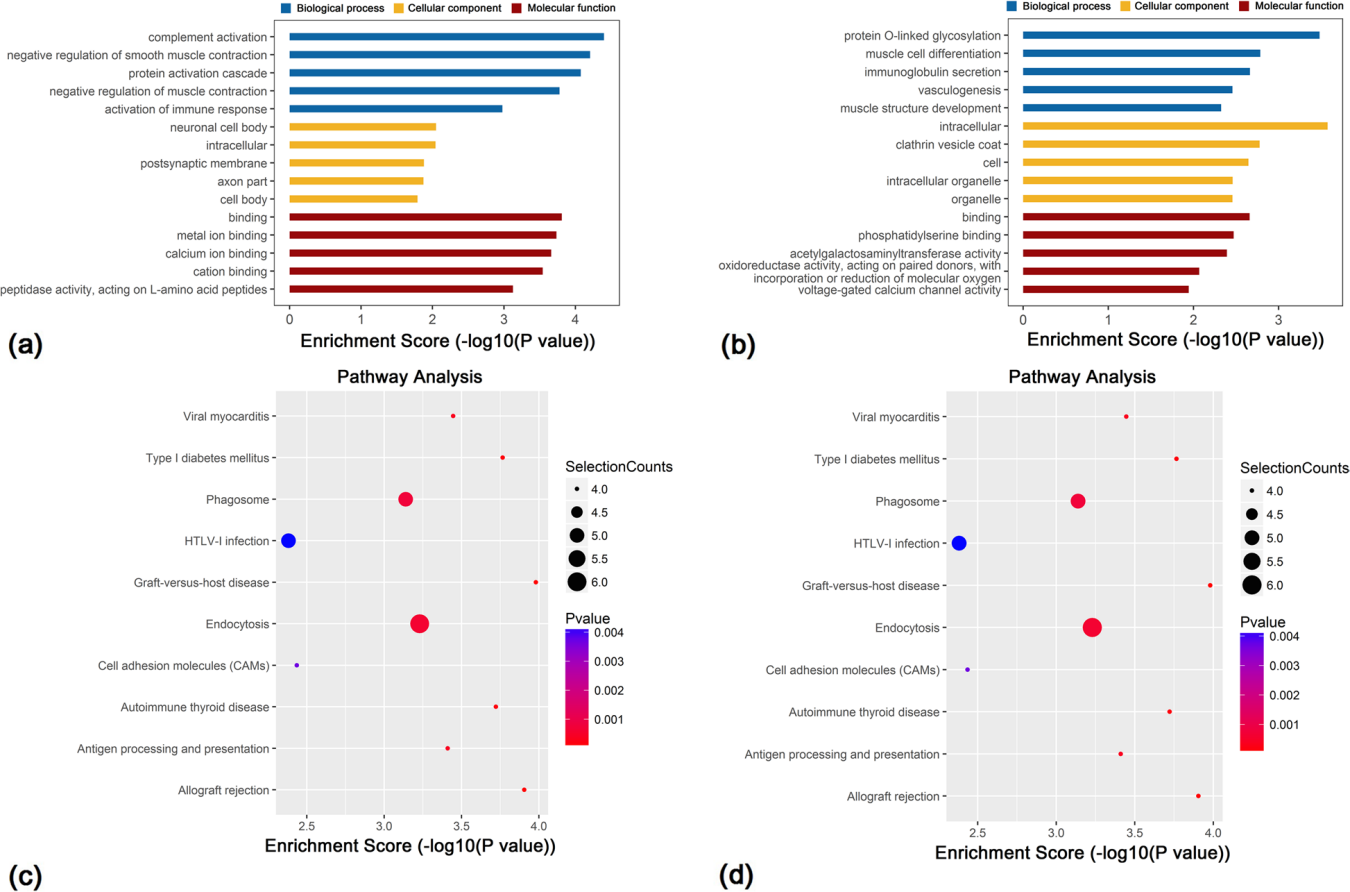
Functional analysis of mRNAs located near differentially- methylated lncRNAs. GO enrichment analysis of genes located near m7G (**a**) hypermethylated and (**b**) hypomethylated lncRNAs. GO enrichment analysis included BP, CC and MF analyses. KEGG pathway analysis of genes located near m7G (**c**) hypermethylated and (**d**) hypomethylated lncRNAs. *P*-values were calculated using DAVID. Selection counts refer to the number of selected genes overlapping with differentially methylated lncRNAs in that pathway. n = 3 for each group

In the biological process (BP) category, GO results revealed that genes near hypermethylated m7G lncRNAs primarily participated in complement activation, negative regulation of smooth muscle contraction and the protein activation cascade. In the cellular component (CC) category, genes were primarily associated with the neuronal cell body, intracellular cellular component and postsynaptic membrane. In terms of molecular function (MF), genes were primarily involved in binding, particularly metal and calcium ion (Fig. 3a). Genes located near hypomethylated lncRNAs were primarily involved in the following BPs: protein O-linked glycosylation, muscle cell differentiation and immunoglobulin secretion. In terms of CC, genes were associated with intracellular organelle, the clathrin vesicle coat and cell cellular component. In terms of MF, genes primarily participated in binding, phosphatidylserine binding and acetylgalactosamine transferase activity (Fig. 3b).

KEGG pathway analysis revealed the four most important signalling pathways associated with genes located near hypermethylated lncRNAs were associated with *Staphylococcus aureus* infection, the cGMP-PKG signalling pathway, complement and coagulation cascades, and Fc gamma R-mediated phagocytosis (Fig. 3c). The four most important signalling pathways for genes located near hypomethylated lncRNAs were associated with graft-versus-host disease, type 1 diabetes mellitus, autoimmune thyroid disease and viral myocarditis (Fig. 3d).

These results suggested that m7G-modified lncRNAs may affect the occurrence and development of HPH via BPs, cellular composition, MF and signalling pathways.

### RNA-seq analysis of differentially expressed lncRNAs in HPH

**Fig. 4:**
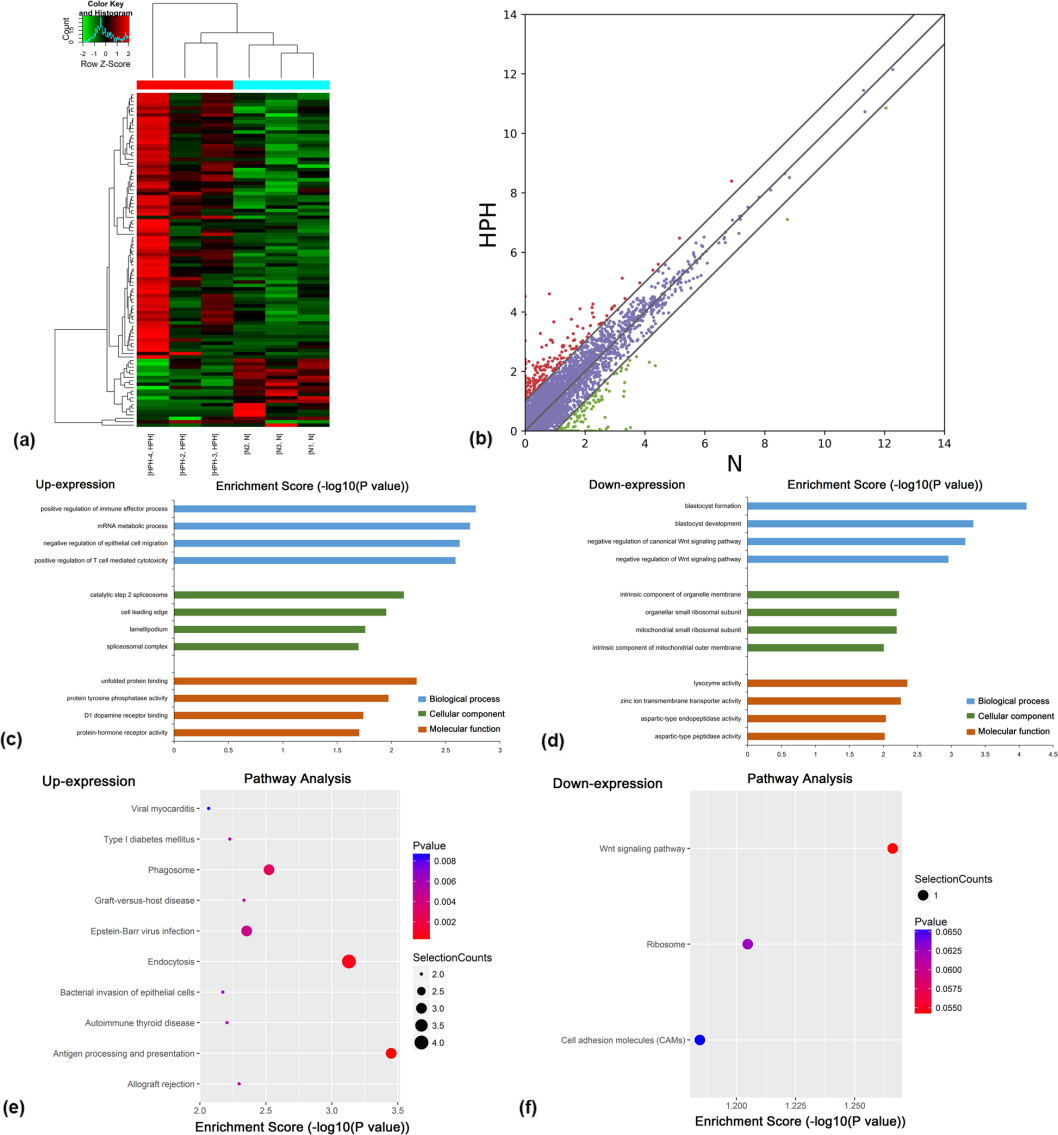
Identification of differentially expressed lncRNAs in lungs of HPH rats compared to normobaric normoxic rats. **a** Heatmap of RNA sequencing data from the two groups. Red, green and black indicate up-regulation, unchanged expression and down-regulation of lncRNAs, respectively. **b** Scatterplot of RNA sequencing data. GO enrichment analysis of genes located near (**c**) up-regulated and (**d**) down-regulated lncRNAs. GO enrichment analysis included BP, CC and MF analysis. KEGG pathway analysis of genes located near (**e**) up-regulated and (**f**) down-regulated lncRNAs. P-values were calculated using DAVID. Selection Counts indicate the number of selected genes that overlap with differentially methylated lncRNAs in that pathway. n = 3 for each group

Figure 4a shows a heatmap of RNA-seq data in the HPH and control groups, indicating the differential expression of lncRNAs in the two groups. A total of 111 lncRNAs were differentially expressed in HPH, including 90 up-regulated and 21 down-regulated types (Fig. 4b).

To further analyse the effects of genes located near differentially expressed lncRNAs, GO enrichment and KEGG pathways analysis of genes near lncRNAs were performed. GO analysis revealed that genes located near up-regulated lncRNAs were significantly enriched in terms of positive regulation of immune effector processes, catalytic step 2 spliceosome and unfolded protein binding (Fig. 4c). Genes near down-regulated lncRNAs were significantly associated with blastocyst formation, lysozyme activity and intrinsic components of organelle membranes (Fig. 4d).

KEGG pathway analysis revealed that genes near up-regulated lncRNAs were primarily involved in antigen processing and presentation, endocytosis, and phagocytosis (Fig. 4e). Genes located near down-regulated lncRNAs were mainly involved in the Wnt signalling pathway. Together, these results indicated that genes near differentially expressed lncRNAs may be related to HPH (Fig. 4f).

### Association of m7G methylation and the expression of lncRNAs

**Fig. 5:**
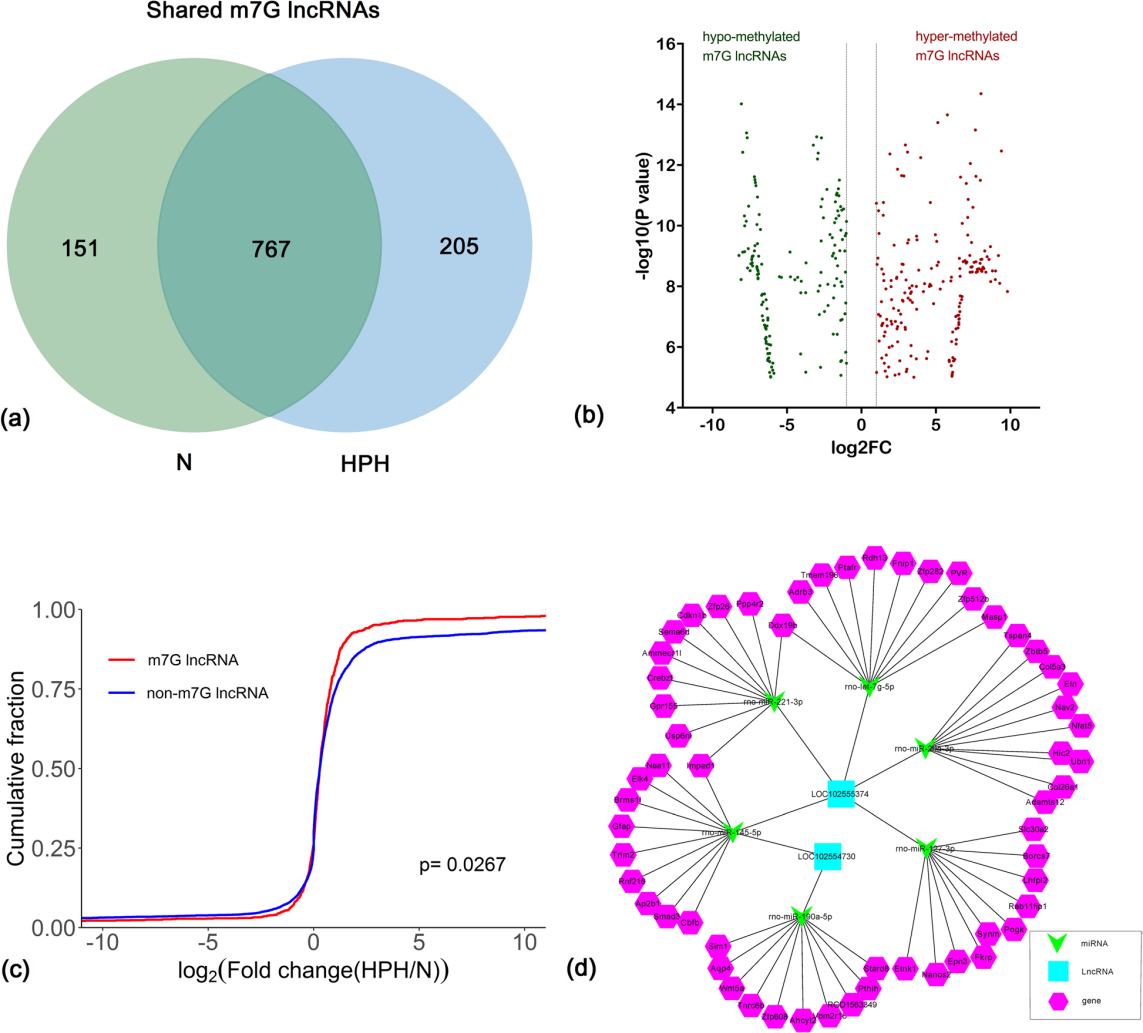
The association between lncRNA m7G methylation and expression. **a** Venn diagram depicting the number of HPH-unique, normoxic (N)-unique, and common m7G lncRNAs. **b** Identification of hypermethylated and hypomethylated m7G lncRNAs showing a significant increase or decrease in abundance (fold change > 2, *p* < 0.00001), respectively, in HPH samples compared with N samples. FC, Fold Change. **c** Cumulative distribution of lncRNA expression including m7G lncRNAs (red) and non-m7G lncRNAs (blue) between HPH and N groups. **d** The network of lncRNA-miRNA-mRNA regulation in HPH. n = 3 for each group

A total of 972 m7G lncRNAs were identified in the HPH group and 918 m7G lncRNAs were identified in the control group. Among these, 767 m7G-modified lncRNAs were detected in both groups (Fig. 5a). The expression of 767 common m7G lncRNAs was investigated to analyse the effect of hypoxia-regulated m7G methylation. As shown in Fig. 5b, 189 hyper-methylated and 155 hypo-methylated m7G lncRNAs were found in the HPH group compared with the control group (fold change > 2, *P* < 0.0001).

To further determine whether m7G methylation affected lncRNA expression, all the expressed lncRNAs were divided into m7G and non-m7G categories. The log two-fold change (log2FC) values of these lncRNAs were determined and a cumulative curve was generated. The proportion of m7G-modified lncRNAs was larger than that of non-m7G lncRNAs. These results indicated that m7G significantly up-regulated the expression of lncRNAs under hypoxic conditions (Fig. 5c, *P* = 0.0267).

The combined results of methylation sequencing and RNA sequencing revealed that only two up-regulated lncRNAs were hypermethylated, which originated from chr4: 7450738–7,450,981 (LOC102554730, XR_591973) and chr13: 95420341–95,420,800 (LOC102555374, XR_592398). However, no down-regulated lncRNAs were hypomethylated.

### Construction of lncRNA-miRNA-mRNA competing endogenous RNA (ceRNA) network and identification of differentially expressed m7G lncRNAs

To explore the potential mRNAs, which were regulated by the lncRNAs, lncXR_592398 and lncXR_592398 were used to build a ceRNA network. Among the predicted miRNAs, miR-190a-5p, miR-145-5p, miR-29a-3p, let-7 g-5p, miR-127-3p and miR-221-3p were selected to construct the ceRNA network because they were related to PH [23]. The top 10 genes for each of these miRNAs were predicted using MirTarget and Miranda programs (Table 1). Thus, the network consisted of PH-associated miRNAs combined with the two differentially expressed m7G lncRNAs and the top 10 mRNAs that bound to the miRNAs, including two lncRNAs, six miRNAs and 58 mRNAs. This ceRNA network clearly suggests that lncRNAs regulated miRNAs and mRNAs. For example, LOC102555374 (XR_592398) bound to rno-let-7 g-5p to regulate Tmem198 expression (Fig. 5d).

**Table 1:** List of downstream target genes of differentially expressed m7G lncRNAs via ceRNA network analysis

lncRNA	miRNAs	Target genes
LOC102555374	rno-let-7 g-5p	Ptafr, Masp1, Tmem198b, Fnip1, Zfp512b, Zfp282, PVR, Ddx19b, Rdh13, Adrb3
LOC102555374	rno-miR-127-3p	Etnk1, Borcs7, Lhfpl2, Synm, Fkrp, Epn3, Slc30a2, Pogk, Rab11fip1, Nanos2
LOC102555374	rno-miR-221-3p	Ddx19b, Impad1, Crebzf, Gpr155, Zfp26, Usp6n1, Ammecr11, Sema6d, Ppp4r2, Cdkn1b
LOC102555374	rno-miR-29a-3p	Adamts12, Col26a1, Zbtb5, Nfat5, Nav2,Hic2, Col5a3, Eln, Tspan4, Ubn1
LOC102555374 LOC102554730	rno-miR-145-5p	Trim2, Ap2b1, Brms11, Gfap, Cbfb, Rnf216, Impad1, Naa11, Smad3, Elk4
LOC102554730	rno-miR-190a-5p	Tnrc6b, Vom2r18, RGD1563349, Aqp4, Wnt5a, Ahcyl2, Sim1, Stard8, Pth1h, Zfp608

### Up-regulation of m7G XR_591973 and m7G XR_592398 in hypoxic PASMCs

**Fig. 6:**
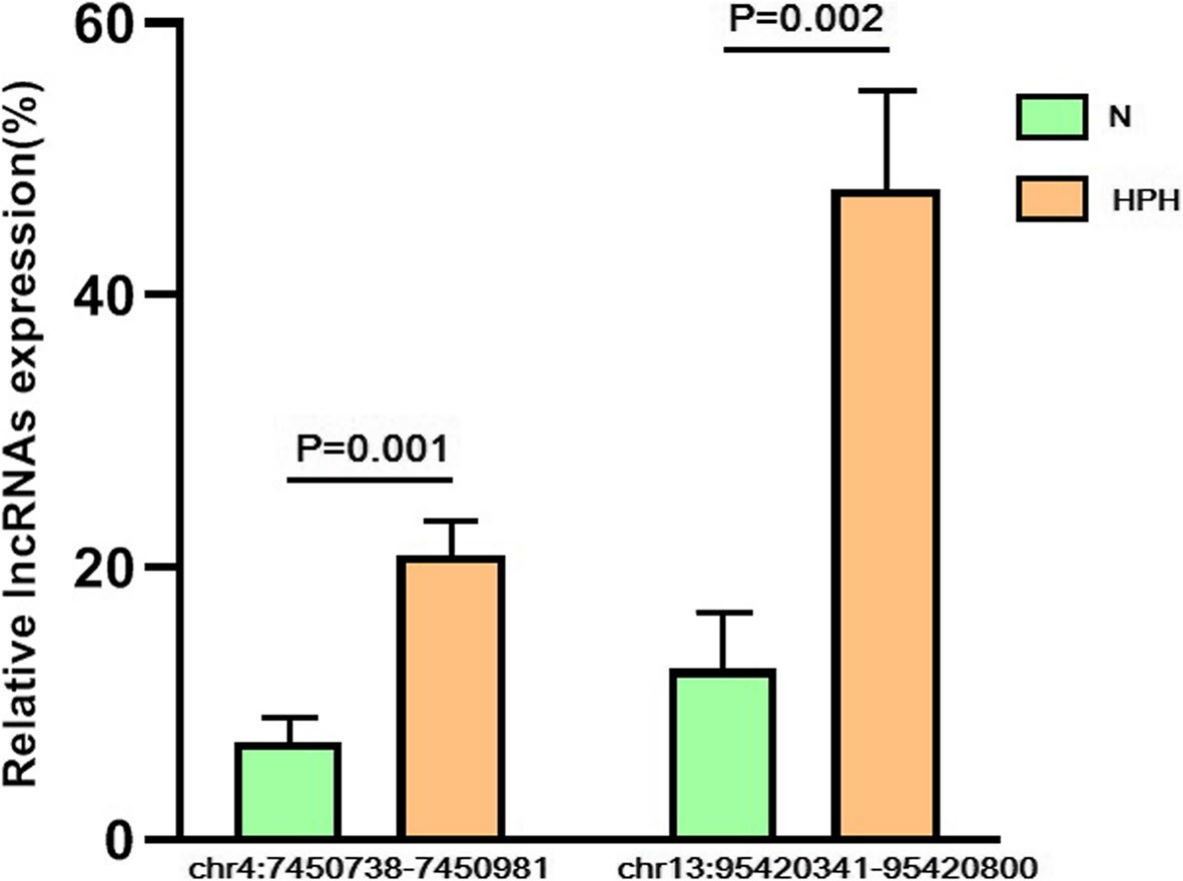
The expression of m7G lncXR_591973 and m7G lncXR_592398 in PASMCs in hypoxia. MeRIP-qRT-PCR detection of differentially expressed m7G lncRNAs. qRT-PCR was performed after MeRIP in PASMCs exposed to 21% (N) or 1% O_2_ (H) for 48 h. The expression of each m7G lncRNA was calculated as the ratio of the anti-m7G level (IP) relative to the control level (input). Data are expressed as the mean ± SD (n = 3 in each group). The P-value was determined using a two-sided paired t-test

To further corroborate the results, the expression of the two differentially expressed m7G lncRNAs in PASMCs treated with hypoxia (1% O_2_) and normoxia (21% O_2_), respectively, was validated. Using cDNA from the PASMCs as templates, lncXR_591973 and lncXR_592398 were amplified by divergent primers. MeRIP-qRT-PCR was used to determine whether lncXR_591973 and lncXR_592398 were modified by m7G. As shown in Fig. 6, the levels of m7G lncXR_591973 and m7G lncXR_592398 were significantly increased in hypoxic PASMCs (*P* = 0.001 and *P* = 0.002, respectively), demonstrating the reliability of the transcriptome-wide m7G-MeRIP-seq analysis.

## Discussion

In the present study, transcriptome-wide mapping of m7G lncRNAs in HPH was performed for the first time to elucidate the role of m7G lncRNAs in the pathogenesis of PH. The m7G modification pattern in HPH samples was distinct from that of normal controls, with a tendency towards lower total m7A abundance in the HPH group. In addition, the two groups shared only 5% m7G peaks. A recent study found that internal m7G/G levels were quite low (range from ~ 0.02% to ~ 0.05%) in human and mouse cell lines within mammalian mRNA, which represent roughly 5–10% of their respective m6A/A ratios. Further, m7G was detected internally within mammalian mRNA. Although the m7G MeRIP-seq method has been widely used, the method still has limitations in detecting m7G internally [24]. These limitations might result in the minor overlap in m7G peaks found in this study.

The transcriptome-wide analysis revealed that m7G modifications were associated with a positive correlation with lncRNA expression in HPH samples. This result was further corroborated by m7G MeRIP-qRT-PCR assays. Similarly, the abundance of m6A modification has been positively associated with lncRNA expression [25]. Kouzarideset al. recently reported that m7G modifications promoted human miRNA processing by directly affecting the secondary structure of pri-miRNAs, which suppressed cell migration [26]. Additionally, m7G modification was also demonstrated to be essential for efficient pre-mRNA splicing, translation and protein-RNA interactions [27]. Further, accumulating evidence suggests that m7G modification is significantly related to the development of multiple diseases, including cancers, infections and stem cell-associated diseases [28].

The KEGG analysis in the present study revealed a significant role of up-regulated m7G lncRNAs in vascular smooth muscle contraction pathway. Pulmonary vasoconstriction is known to play an important role in the pathogenesis of HPH and represents one of the factors contributing to increased PASMC contraction [4]. Clinical studies have also shown that vasodilating agents ameliorate PH and cardiac output of patients diagnosed with HPH, which demonstrates the key role of vasoconstriction in HPH pathophysiology [29, 30]. A previous study further reported that inhibiting PASMC contraction in response to hypoxia significantly decreased the RVP of PH models [31].

lncRNAs act as ceRNAs to indirectly regulate mRNAs via shared microRNAs, which represents a novel layer of RNA crosstalk and plays a critical role in the development of various diseases [32]. m7G plays a critical role in regulating RNA processing, metabolism and function [28], suggesting that m7G also regulates the lncRNA-miRNA-mRNA co-expression network. Analysis of the differentially expressed m7G lncRNAs revealed an overlap of 11 pseudogenes between the identified lncRNAs. Among the overlapped pseudogenes, Smad3 is a key downstream element in transforming growth factor (TGF)-β signaling pathways [33]. In recent years, the critical role of TGF-β signaling pathways in PH has been better elucidated. Enhanced TGF-β signaling has been associated with PH accompanied by smooth muscle hypertrophy, perivascular fibrosis, and extracellular matrix remodeling [34]. Another overlapped gene, inositol monophosphatase domain containing 1(Impad1), a novel sulfotransferase that is normally located in golgi bodies, has received increased attention in cancer, as it converts PAP into AMP in human fibroblasts [35]. Increasing evidence indicates that in cancer, Impad1 acted in the ER-Golgi pathway by altering Golgi-mediated secretion of MMPs, resulting in a pro-invasive and metastatic tumour phenotype [36]. Additionally, Impad1 overexpression was shown to inhibit Complex I activity, reducing ROS production in cancer cells, and promoting tumor cell invasion [37]. As a result, the function of Impad1 is diverse and needs further exploration in HPH. Among the non-overlapped pseudogenes, nuclear factor of activated T cells 5 (Nfat5), the Ca2+ / calcineurin sensitive transcription factor, was reported to perpetuate the elevation in cytosolic calcium and promote apoptosis resistance of PASMCs. Increased cytosolic calcium contributes to the contractile, hyperproliferative, and anti-apoptotic phenotype of PASMCs, which leads to PH pathogenesis [38]. However, Wnt5a, a key factor of Wnt signaling pathways, plays a key role in the Wnt signaling pathways in PAH pathobiology. Evidence suggests that alterations in Wnt pathway activation lead to PH by preventing proper small vessel regeneration and allowing excessive PASMC growth [39]. In general, these downstream targets regulated by lncRNA-miRNA of interest were mostly enriched in PH-associated TGF-β, Wnt and Nfat signaling pathways. Our study reported for the first time that m7G influenced lncRNAs, thus regulating the binding of miRNA, resulting in the activation of TGF-β, Wnt and Nfat signaling pathways.

The main limitations of this study were the lack of clinical samples and the inability to determine the precise mechanism of hypoxia-induced m7G modification. Thus, studies focusing on the underlying mechanism of m7G modification, as well as clinical transformation, are required to establish the role of m7G lncRNAs in the pathogenesis of HPH.

## Conclusion

This study provides the first m7G transcriptome-wide map of HPH. It demonstrated that the m7G modification pattern in HPH samples is distinct from that of controls, with a tendency towards lower total m7A abundance in the HPH group. Further, two significant m7G lncRNAs were found to be associated with HPH for the first time, and both of them are validated in PASMC cells. The role of m7G lncRNAs in HPH and its underlying mechanism require further exploration. It is hoped that this will be the start of studies investigating m7G functions and modification in HPH. In the future, the integrated analysis of transcription and translation studies might contribute to the elucidation of the mechanism of HPH. Additional evidence derived from large patient populations with HPH will improve the value of this study and identify specific HPH-related m7G methylation sites.

## Methods

### Establishment of HPH rat model

4-week male Sprague-Dawley (SD) rats weighing 180 to 200 g each were used in this study and were supplied by the Animal Experimental Centre, Zhejiang University, China. Rats were randomly allocated to the HPH and the control group (*n* = 6 in each group). The rat model for this study was established based on our previous study [22], and the same animal group was used in the two studies. The construction and subsequent verification of a hypoxia-induced PH rat model has been described in our previous study [22]. All rats were sacrificed by exposure to 100% CO_2_ in a confined and transparent euthanasia device, and heart and lung tissues were removed for the following experiments. All protocols and procedures were approved by the Ethics Committee of Zhejiang University, China and were conducted in accordance with the guidelines of the National Institutes of Health on the care and use of animals.

### Isolation and hypoxia treatment of primary PASMCs

The isolation of primary PASMCs from rat pulmonary artery was performed as described previously [17]. PASMCs were cultured in Dulbecco’s modified Eagle medium supplemented with 10% foetal bovine serum (FBS). Before experimental use, the cells were incubated under hypoxic (1% O_2_) or normoxic (21% O_2_) conditions at 37 °C for 48 h. PASMCs were grown to no more than 90% confluence and used between passages four and seven.

### RNA isolation and RNA-seq analysis

Total RNA from lungs (1 g) of control and HPH rats was extracted with TRIzol reagent (Invitrogen, CA, USA). The extracted RNA was purified using a NEBNext rRNA Depletion Kit (New England Biolabs, MA, USA). Next, RNA libraries were prepared using the NEBNext Ultra Directional RNA Library Prep kit (New England Biolabs, MA, USA). Library sequencing was performed on an IlluminaHiseq instrument with 150 bp paired-end reads.

### MeRIP and m7G-seq analysis

The isolated RNA was subjected to immunoprecipitation using the m7G-IPKit (GenSeq, Beijing, China) according to the manufacturer’s instructions. RNA was randomly fragmented to about 200 nt using RNA fragmentation reagents (Millipore Sigma, USA). Protein A/G beads (Thermo Fisher Scientific, USA) were coupled to the m7G antibody via rotation at room temperature for 1 h. The RNA fragments were incubated with the bead-linked antibodies and rotated at 4 °C for 4 h. Then, the bound RNA was eluted from the beads using Proteinase K and purified by phenol-chloroform extraction. Purified RNA was used for RNA-seq library generation with a NEBNext® Ultra II Directional RNA Library Prep Kit (New England Biolabs, MA, USA). Paired-end reads were harvested from an IlluminaHiSeq 4000 sequencer.

### Construction of a ceRNA network

The lncRNA-microRNA (miRNA) -mRNA ceRNA network was constructed based on the theory that lncRNA acts as an miRNA sponge to further regulate mRNA. The binding sites of miRNA on differentially expressed m7G lncRNAs as well as the target mRNA of the miRNA were predicted with Target Scan and miRanda. The miRNAs related to PH and the top 10 mRNAs binding to each selected miRNA were retained for further network analysis. Pearson correlation analysis was used to assess the ceRNA network construction. Cytoscape v3.6.0 was used to visualise the ceRNA network.

### Methylated RNA immunoprecipitation and MeRIP-qPCR

M7G immunoprecipitation (MeRIP) was performed to measure the specific m7G modification of lncRNA. Total RNA was isolated from hypoxia- and normoxia-treated PASMCs. RNA (18 μg) was fragmented to approximately 200 nt-long fragments in an RNA fragmentation buffer. The fragmented RNA was purified using an RNaseMiniElute Kit according to the manufacturer’s instructions. Protein A/G beads (50 μL) were coupled to m7G antibody by rotating at room temperature for 30 min. The RNA fragments were then incubated with the bead-linked antibodies and rotated at 4 °C for 2 h. The immunopurified RNA was purified and first-strand cDNA synthesis was performed by real-time quantitative PCR (QPCR). All the samples were run with the qPCR SYBR Green Master Mix (CloudSeq). The primers used for the detection of m7G-enriched lncRNA are presented in Table 2.

**Table 2:** Primer sequences used in the qPCR analysis

Gene (GenBank)	Primer sequence (5′-3′)
chr4:7450738–7,450,981	Forward GTGATGTGGAAGGGAGCACT
	Reverse TGTCTGCCTCTCCGTCTTCT
chr13:95420341–95,420,800	Forward AGGACACCAAGGGAACACTG
	Reverse CAGGTACCTCCCAAGCCATA

### Sequencing data analysis and statistical analysis

The quality of harvested paired-end reads were controlled by Q30. Cutadapt (v1.9.3) was used for 3′ adaptor trimming and the removal of low-quality reads. Clean reads from all libraries were aligned with the reference genome (UCSC RN5) using HISAT2 (v2.0.4). The m7G MeRIP-sequencing protocol was performed using methylated sites on RNAs (m7G peaks) identified by MACS software. Differentially methylated sites were identified by diffReps and m7G peaks that overlapped with transcription exons were retained for further study. For lncRNA sequencing, the FPKM (fragments per kilobase of exon per million fragments mapped) value was obtained as the expression profiles of lncRNA using Cuffdiff (v2.2.1). Differentially expressed lncRNAs were identified by the fold change and *p*-value (fold change ≥2 and *P* ≤ 0.00001). In addition, gene ontology (GO) and kyoto encyclopedia of genes and genomes (KEGG) pathway enrichment analysis of the differentially-expressed RNAs and the differentially-methylated protein-coding genes were performed according to previous reports [40, 41]. *P* values are calculated by DAVID tool for GO and KEGG pathway analysis.

Data were presented as the mean ± standard deviation (SD). Statistical analyses were performed using SPSS 19.0 (Chicago, IL, USA) and GraphPad Prism 5.0 (La Jolla, CA). A two-tailed Student’s t-test or two-sided Wilcoxon-Mann-Whiteney test was used to determine the significant differences between the two groups. The number of samples in each group is shown as n in figure legends. Differences of *P* < 0.05 were defined as statistically significant. All experiments were independently repeated at least three times.

## Supplementary Information

**Additional file 1: Data S1**: m7G methylated sites of lncRNAs in HPH and control samples.

**Additional file 2: Data S2**: Differentially expressed m7 Gmethylated sites of lncRNAs in the HPH rat model compared to the control rat model.

## Data Availability

The raw high-throughput m7G and lncRNAs sequencing generated during the current study can be obtained from the NCBI Gene Expression Omnibus (GEO): accession number GSE182042 and GSE182054, [https://www.ncbi.nlm.nih.gov/geo/query/acc.cgi?acc=GSE182042], token:[klsluyqezjkfdir]. [https://www.ncbi.nlm.nih.gov/geo/query/acc.cgi?acc=GSE182054], token:[kvmxqquixvszrmj].

## Abbreviations

lncRNAs: Long non-coding RNAs
HPH: Hypoxic pulmonary hypertension
m7G: N7-methylguanosine
PH: Pulmonary hypertension
COPD: Chronic obstructive pulmonary disease
PAECs: Pulmonary artery endothelial cells
PASMCs: pulmonary artery smooth muscle cells
BP: Biological process
CC: Cellular component
MF: Molecular function
SD: Sprague-Dawley
FBS: Foetal bovine serum
miRNA: microRNA
SD: Standard deviation
GO: Gene ontology
KEGG: Kyoto encyclopedia of genes and genomes
Wnt5a: Wnt family member 5a
RGD1563349: Arginine-glycine-aspartic acid1563349
Vom2r18: vomeronasal 2 receptor, 18
Zfp512b, Zfp282,Zfp608, Zfp26: zinc finger protein 512b, 282, 608 and 26
Smad3: SMAD family member 3
Impad1: Inositol monophosphatase domain containing 1
Ap2b1: Adaptor related protein complex 2 subunit beta 1
Cbfb: Core-binding factor subunit beta
Naa11: N-alpha-acetyltransferase 11
Brms1l: Breast cancer metastasis-suppressor 1-like
Tspan4: Tetraspanin 4
Usp6nl: USP6 N-terminal like
Pthlh: Parathyroid hormone like hormone
Col5a3: Collagen type V alpha 3 chain
Adrb3: Adrenoceptor beta 3
Nfat5: Nuclear factor of activated T cells 5
Nanos2: Nanos C2HC-type zinc finger 2
Sema6d: Semaphorin 6D
Tnrc6b: Trinucleotide repeat containing adaptor 6B
Slc30a2: Solute carrier family 30 member 2
Masp1: MBL associated serine protease 1
Fkrp: Fukutin related protein
Rnf216: Ring finger protein 216
Epn3: Epsin 3
Stard8: StAR related lipid transfer domain containing 8
Synm: Synemin
Hic2: HIC ZBTB transcriptional repressor 2
Cdkn1b: cyclin dependent kinase inhibitor 1B
Rdh13: Retinol dehydrogenase 13
Ammecr1l: AMME chromosomal region gene 1-like
PVR: Poliovirus receptor
Pogk: Pogo transposable element derived with KRAB domain
Zbtb5: Zinc finger and BTB domain containing 5
Sim1: Single-minded family bHLH transcription factor 1
Gfap: Glial fibrillary acidic protein
Trim2: Tripartite motif containing 2
Borcs7: BLOC-1 related complex subunit 7
Ahcyl2: Adenosylhomocysteinase like 2
Ptafr: Platelet-activating factor receptor
Fnip1: Folliculin interacting protein 1
Gpr155: G protein-coupled receptor 155
Adamts12: A disintegrin-like and metallopeptidase (reprolysin type) with thrombospondin type 1 motif, 12
Ubn1: ubinuclein 1
Rab11fip1: RAB11 family interacting protein 1
Tmem198b: Transmembrane protein 198b
Aqp4: Aquaporin 4
Crebzf: CREB/ATF bZIP transcription factor
Lhfpl2: lipoma HMGIC fusion partner-like 2
Etnk1: Ethanolamine kinase 1
Col26a1: Collagen type XXVI alpha 1 chain
Ppp4r2: Protein phosphatase 4 regulatory subunit 2
Ddx19b: DEAD-box helicase 19B
Eln: Elastin
Nav2: Neuron navigator 2
